# Effects of the intensity, duration and muscle mass factors of isometric exercise on acute local muscle hemodynamic responses and systematic blood pressure regulation

**DOI:** 10.3389/fbioe.2024.1444598

**Published:** 2024-08-01

**Authors:** Songmei Lin, Pu Sun, Liwan Huang, Manuel Hernandez, Hongjun Yu, Yih-Kuen Jan

**Affiliations:** ^1^ Department of Kinesiology and Community Health, University of Illinois at Urbana-Champaign, Urbana, IL, United States; ^2^ College of Physical Education and Sports, Beijing Normal University, Beijing, China; ^3^ Department of Biomedical and Translational Sciences, University of Illinois at Urbana-Champaign, Urbana, IL, United States; ^4^ Department of Physical Education, Tsinghua University, Beijing, China

**Keywords:** dose response, ischemia, isometric contraction, near infrared spectroscopy, blood pressure

## Abstract

Isometric exercise is a non-pharmacologic intervention to improve muscle hemodynamic responses and blood pressure in humans. However, the effects of intensity, duration, and muscle mass factors of isometric exercise on local muscle hemodynamic responses and systemic blood pressure regulation have not been studied. The purpose of this study was to assess whether various modes of isometric exercise could induce various levels of muscle hemodynamic responses that are related to the blood pressure changes. Near-infrared spectroscopy was used to assess muscle hemodynamic responses after 4 isometric exercise protocols in 20 healthy adults. One-way analysis of variance (ANOVA) with repeated measures was used to assess the effect of factors of isometric exercise on oxyhemoglobin, deoxy-hemoglobin, blood volume, and oxygenation. For oxygenation, the lowest mean was recorded for the unilateral isometric handgrip exercise at 30% of MVC for 2 min (−0.317 ± 0.379 μM) while the highest mean was observed for the isometric wall squat (1.496 ± 0.498 μM, *P* < 0.05). Additionally, both the bilateral isometric handgrip exercise at 30% MVC for 1 min (1.340 ± 0.711 μM, *P* < 0.05) and the unilateral isometric handgrip exercise at 20% MVC for 3 min (0.798 ± 0.324 μM, *P* < 0.05) are significantly higher than 30% of MVC for 2 min. Blood pressure showed an inverse trend with oxygenation changes of the forearm muscle. The study indicates that the duration and muscle mass of isometric exercise are more effective on oxygenation responses and systematic blood pressure regulation, and suggests that the local muscle oxygenation factor following isometric contractions may mediate systematic blood pressure regulation.

## Introduction

Isometric exercise is a popular exercise because people can easily perform various isometric exercise, such as handgrip exercise and wall squat, without using expensive equipment and dedicated space (Lawrence et al., 2015). Isometric exercise is endorsed by the American College of Cardiology and the American Heart Association as a non-pharmacological intervention to manage hypertension (Whelton et al., 2018). A meta-analysis indicates that simple resistance training, such as isometric handgrip training, could significantly reduce blood pressure in people with and without hypertension (Cornelissen et al., 2011). Isometric exercise usually consists of four 2-min sessions at 30% of MVC with short breaks in between (McGowan et al., 2017), three times a week for 8–10 weeks (McGowan et al., 2017; Whelton et al., 2018). Early evidence suggests that isometric exercise reduces 24-h ambulatory blood pressure (Somani et al., 2017). Isometric exercise has been shown to lower blood pressure in a shorter time period compared with aerobic exercise (Cornelissen et al., 2011). Although isometric exercise has been shown to benefit blood pressure management, mixed results of the outcomes of isometric exercise training have been reported (Chrysant, 2010; Inder et al., 2016; Jin et al., 2017). Due to the heterogeneity of the results reported in these studies, uncertainty remains regarding the efficacy of isometric contraction training on reduction of systolic, diastolic, and mean arterial pressures (Inder et al., 2016; Jin et al., 2017).

The inconsistent results may be partly attributed to different intensities and durations of isometric exercise used in these trials. The intensity of isometric exercise is usually measured as a percentage of maximal voluntary contraction (MVC) and the duration is to maintain this intensity through isometric exercise (Ogbutor et al., 2022). In young normotensive adults, an isometric exercise training regimen consisting of 4 2-min contractions at 30% of MVC, 3 days per week for 5 weeks resulted in a slightly higher reduction in systolic blood pressure than 20% of MVC in a similar group, and the difference in diastolic blood pressure reduction was not significant (Howden et al., 2002). Some isometric exercise training studies used a 4 2-min isometric handgrip contraction, 3 days per week for 8 weeks, and found an improvement in blood pressure (Millar et al., 2007; Kelley and Kelley, 2010), whereas some studies have shown no changes (McGowan et al., 2006; Stiller-Moldovan et al., 2012). A randomized controlled trial found that isometric exercise did not lower blood pressure and only aerobic exercise lowered blood pressure in hypertensive patients (Pagonas et al., 2017). These mixed results could be due to the use of different intensities and durations of isometric exercise because clinical guidelines and the mechanism of action of isometric exercise have not been established.

To date, three potential mechanisms of action have been proposed to explain the effect of isometric contraction on blood pressure regulation, including the cardiac autonomic mechanism (Millar et al., 2014), central and peripheral autonomic regulation of vascular tone (Ray and Carrasco, 2000), and alterations in vascular function (Badrov et al., 2013). The theory based on the cardiac autonomic mechanism indicates that isometric handgrip training may improve cardiac vagal regulation for enhancing blood flow and reducing blood pressure (Millar et al., 2013). Another theory argues that isometric handgrip training may help reduce blood pressure by regulating peripheral sympathetic nerve activity and decreasing the sympathetic outflow in hypertensive patients (Yamada et al., 2022). The other potential mechanism for lowering blood pressure and improving vascular endothelial function may be through increased nitric oxide production and/or bioavailability (Beck et al., 2014). Nevertheless, the exact mechanism by which isometric handgrip training affects blood pressure and cardiovascular changes requires further research. Among these theories, muscle ischemia caused by isometric contraction is involved in all theories used to explain the benefit of isometric contraction on inducing blood pressure regulation. The muscle tension during isometric contraction may occlude blood vessels within the contracting muscle for resulting in muscle ischemia (Beck et al., 2014). After isometric contraction, the tissue ischemia may stimulate a vasodilatory response for overcoming the tissue ischemia (Jan et al., 2013; Beck et al., 2014). This may partly explain the reason that effect from isometric contraction is different from both concentric and eccentric contraction without causing constant muscle tension for tissue ischemia. Nevertheless, the relationship between muscle hemodynamic responses and blood pressure has not been fully investigated.

Muscle hemodynamic response to isometric exercise can be assessed by technologies, including venous occlusion volume tracing (VOP), functional magnetic resonance imaging (fMRI), positron emission tomography (PET), and near-infrared spectroscopy (NIRS) (McNeil et al., 2015). Among these technologies, NIRS provides a real-time, simultaneous examination of changes in oxyhemoglobin and deoxy-hemoglobin of the muscle (Bhambhani, 2012; Kruse et al., 2016), and could be a promising tool for studying the effect of local muscle blood volume and oxygenation changes induced by isometric exercise on systematic blood pressure regulation. Blood volume, defined as the sum of oxyhemoglobin and deoxy-hemoglobin, is also considered an indicator of local tissue blood flow. Oxygenation, defined as the difference between oxyhemoglobin and deoxy-hemoglobin, is also used as an indicator for tissue O_2_ extraction (Orcioli-Silva et al., 2024). For example, McCully found that with intermittent isometric contractions of muscles, a corresponding decrease in muscle oxygenation is observed (McCully, 2010). Additionally, 25% and 35% of MVC of isometric contraction resulted in significant reductions in blood volume and oxygenation in skeletal muscle tissue (De Ruiter et al., 2007). A review indicates that the measured intramuscular oxygenation and blood volume are associated with the isometric resistance training regimen (i.e., exercise intensity, involved muscle groups, and exercise duration) (Pereira et al., 2007). The muscle mass factor has been attributed to the total volume of microvascular vessels, that is, a larger muscle mass may have more blood vessels and when under isometric contraction, these blood vessels would release more vasodilators for inducing a larger hemodynamic response (Li et al., 2024; Mo et al., 2024).

In order to better understand the interaction between local muscle hemodynamic responses and systematic blood pressure regulation during isometric contractions under various intensities, durations and muscle mass, it is needed to use NIRS to measure local oxyhemoglobin and deoxy-hemoglobin responses after isometric exercise and correlate them with systematic blood pressure regulation. Given the potential use of isometric exercise in clinical populations who use wheelchairs and cannot perform regular aerobic exercise, such as running, it is imperative to identify the acute hemodynamic response of the muscles and blood pressure changes after isometric exercise. The primary objective of this study was to compare changes in local oxyhemoglobin, deoxy-hemoglobin, blood volume, and oxygenation and systematic blood pressure after different isometric exercise conditions. We hypothesized that the intensity, duration and muscle mass factors of isometric contractions (handgrip and wall squat) are related to the changes in systematic blood pressure. The finding of this study can help elucidate the relationship between local muscle hemodynamic response and systematic blood pressure changes and establish the dose-response relationship of isometric contraction for improving blood pressure regulation.

## Methods

A repeated-measures study design was used to assess the effects of isometric exercise on the hemodynamic response of muscle. The study design was chosen to minimize the effects of between-subject characteristics in this study. The data collected in this study were coded and blinded to the person performing the data analysis of the NIRS signals. This study is part of a series of studies evaluating muscle hemodynamic responses under various isometric exercise conditions. The research study was approved by the University of Illinois at Urbana Champaign Institutional Review Board (IRB #24355).

## Participants

The inclusion criteria were: age between 18 and 40 years, being able to independently perform isometric handgrip exercise and isometric wall squat. The exclusion criteria included diagnosed diabetes, any vascular diseases, any neuromuscular impairment, smokers, and hypertension. All subjects in this study were recruited from the University of Illinois at Urbana-Champaign through flyers and word of mouth. Subjects were informed of the experimental procedures and the potential risks involved and signed an informed consent form. Subject recruitment took place between 1 February 2024 and 15 April 2024.

## Instrumentation

**FIGURE 1:**
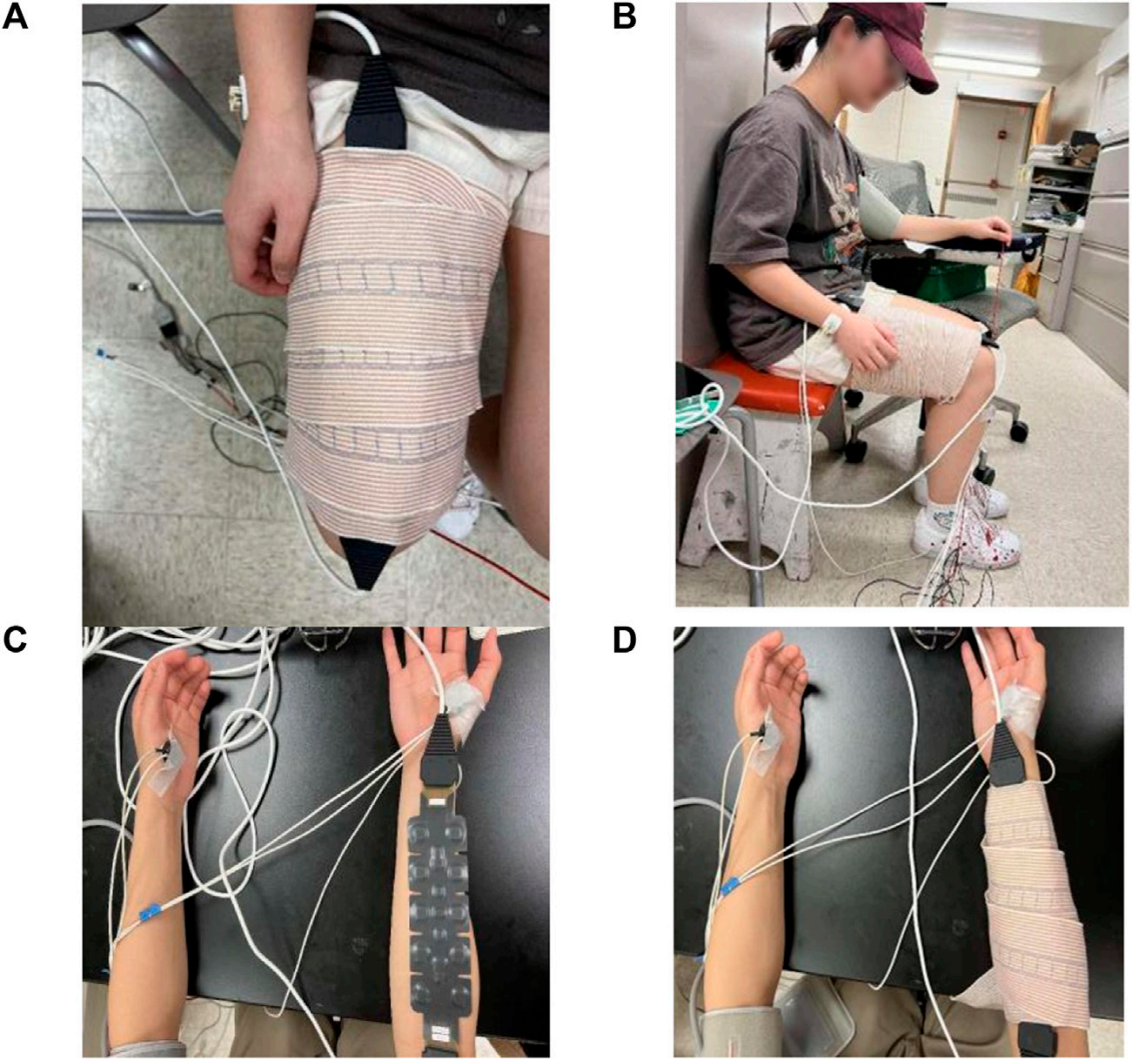
Photographs of NIRS equipment and the setup of the NIRS equipment. **(A)** The placement of NIRS on the thigh. The NIRS sensor is wrapped around the right quadriceps femoris. **(B)** The side view of the thigh with NIRS. **(C)** NIRS sensor is placed on the forearm. **(D)** The NIRS sensor is wrapped around the forearm muscle.

A 16-channel NIRS system (fNIR Imager 1000, fNIR Devices LLC, Potomac, Maryland) was used to measure muscle hemodynamic responses, including the concentrations of oxyhemoglobin and deoxy-hemoglobin. The sensor band includes ten photodetectors and four light emitting diode (LED) lights. NIRS signals were sampled at a frequency of 2 Hz. For the calibration procedures, spectrometer settings were modified according to the participant’s condition to maintain the intensity of the infrared LED to remain within the functional range so as to avoid dark noise levels and saturation (Li et al., 2023; Mo et al., 2024). The raw fNIRS signals were low-pass filtered within a finite impulse response filter with cut-off frequency at 0.14 Hz to eliminate possible respiration and heart rate signals and unwanted high frequency noise. The relative concentration of oxyhemoglobin (Δ[HbO_2_]) and deoxy-hemoglobin) (Δ[Hb] can be calculated by modifying the Bill-Lambert law (Boas et al., 2001). In this study, only signals from the channels 3–14 were used to calculate the muscle hemodynamic responses, and signals from channels 1, 2, 15, and 16 were not used to avoid the edge effect. Systolic and diastolic blood pressure was measured before and immediate post-exercise and 10-min post-exercise of isometric handgrip or wall squat using an automated blood pressure monitoring system (STBP-780, Nihon Colin, Tokyo, Japan) (Figure 1).

## Experimental protocols

In this study, four isometric exercise protocols were investigated including: 1) a common isometric exercise protocol, that is, unilateral isometric handgrip exercise at 30% of MVC for 2 min with 2-min rest for a total of 4 bouts, 2) unilateral isometric handgrip exercise at 20% MVC for 3 min with 2-min rest for a total of 4 bouts, 3) bilateral isometric handgrip exercise at 30% MVC for 1 min with 2-min rest for a total of 4 bouts, and 4) isometric wall squat at 105–110 degrees of knee angle for 1 min with 2-min rest for a total of 4 bouts. The selection of 30% of MVC for 2 min with 2-min rest for a total of 4 bouts was because this is the most common protocol for isometric handgrip exercise (Millar et al., 2007; Kelley and Kelley, 2010). Therefore, we used this protocol as the “standard” for investigating the intensity, duration and muscle mass factors of isometric contraction.

In this study, we defined the exercise volume of isometric exercise as a product of isometric intensity (% of MVC) × isometric duration × isometric muscle mass based on potential factors of isometric exercise. Based ono this definition, all three isometric handgrip exercise protocols have the same exercise volume.

(A) 30% of MVC × 2 min × 1 muscle mass (unilateral) = (B) 20% of MVC × 3 min × 1 muscle mass (unilateral) = (C) 30% of MVC × 1 min × 2 muscle mass (bilateral) = 60% MVC × min × muscle mass.

When comparing (A) and (B), we could investigate the relative efficacy of the intensity factor (A) or the duration factor (B). This would allow us to assess which is a more dominant factor from the intensity and duration factors on modulating blood pressure changes. When comparing (A) and (C), we could investigate the relative efficacy of the duration (A) and muscle mass (C). This would provide relative efficacy on blood pressure from the duration and muscle mass factors. The wall squat (D, muscle mass factor) was used to compare the relative efficacy of lower limb and upper limb isometric exercise. The isometric wall squat was used to assess whether a larger muscle mass from wall squat would induce a more significant change in blood pressure compared to a smaller muscle mass associated with isometric handgrip exercise.

## Experimental procedures

All procedures and examinations were performed at the Rehabilitation Engineering Laboratory at the University of Illinois at Urbana-Champaign. The order of exercise protocols were randomly assigned to the participant to avoid the order effect of tested exercise protocols. The participants were instructed not to pursue exercise and drink alcohol and coffin drink before visiting the lab and rested for 15 min after arrival. The blood pressure monitoring cuff was worn on the participant’s left arm to measure the participant’s blood pressure. The 5-min of baseline data (pre-exercise) of NIRS signals were collected before exercise. The subject performed the maximal grip using the handheld dynamometer for 2 s and rest 1 min for three times. After collecting the data on the three maximum handgrip strength, the average of the three maximum handgrip strength tests was calculated, and then 30% or 20% of the average handgrip strength was calculated for the subsequent isometric protocols. For wall squats, the subject leaned his back on the wall and squatted slowly when the back was in contact with the wall. For the isometric wall squat at 105–110° of knee angle for 1 min with 2-min rest for 4 bouts, the assistant used a protractor to help the subject determine the 105–110° knee angle. Then, a participant sat in a chair for measuring muscle blood flow and blood pressure responses. Subjects were immediately and 10-mint post-exercise tested for blood pressure and muscle hemodynamic response using a sphygmomanometer and NIRS and again at the end of the 10-min flow test. Three blood pressure and two muscle flow responses were measured throughout the procedure. Each subject was asked to complete 4 different experiments on 4 different days.

## Data analysis

Measurements of muscle hemodynamic responses before isometric exercise were used as a baseline to determine changes in oxygenated hemoglobin (Δ[HbO_2_]) and deoxy-hemoglobin (Δ[Hb]) concentrations after isometric exercise. In this study, baseline measurements were taken over a 5-min period. Blood volume was defined as the sum of oxyhemoglobin and deoxy-hemoglobin (Δ[HbO_2_] + Δ[Hb]). Oxygenation was defined as the difference between oxygenated and deoxygenated hemoglobin (Δ[HbO_2_] - Δ[Hb]). A one-way analysis of variance (ANOVA) with repeated measures was used to test for the effect of isometric exercise factors on muscle hemodynamic responses. Distribution tests were performed to determine whether the data were normally distributed. For *post hoc* comparisons, Bonferroni correction was applied. The dependent variables were oxyhemoglobin (Δ[HbO_2_] in μM), deoxy-hemoglobin (Δ[Hb] in μM), blood volume (Δ[HbO2] + Δ[Hb] in μM), and oxygen saturation (Δ[HbO_2_] - Δ[Hb] in μM), and blood pressure. The level of significance was set at *p* < 0.05. All NIRS analyses were performed using MATLAB (2022a, The MathWorks Inc., Natick, MA), and statistical analyses were performed using SPSS (version 29, IBM Corp., Armonk, NY).

## Results

Twenty healthy participants (11 females, 9 males) participated in this study, characterized by (mean ± S.D.): age 24.0 ± 3.5 years, body mass index 23.0 ± 2.8 kg/m^2^. All participants were right-handed.

**FIGURE 2:**
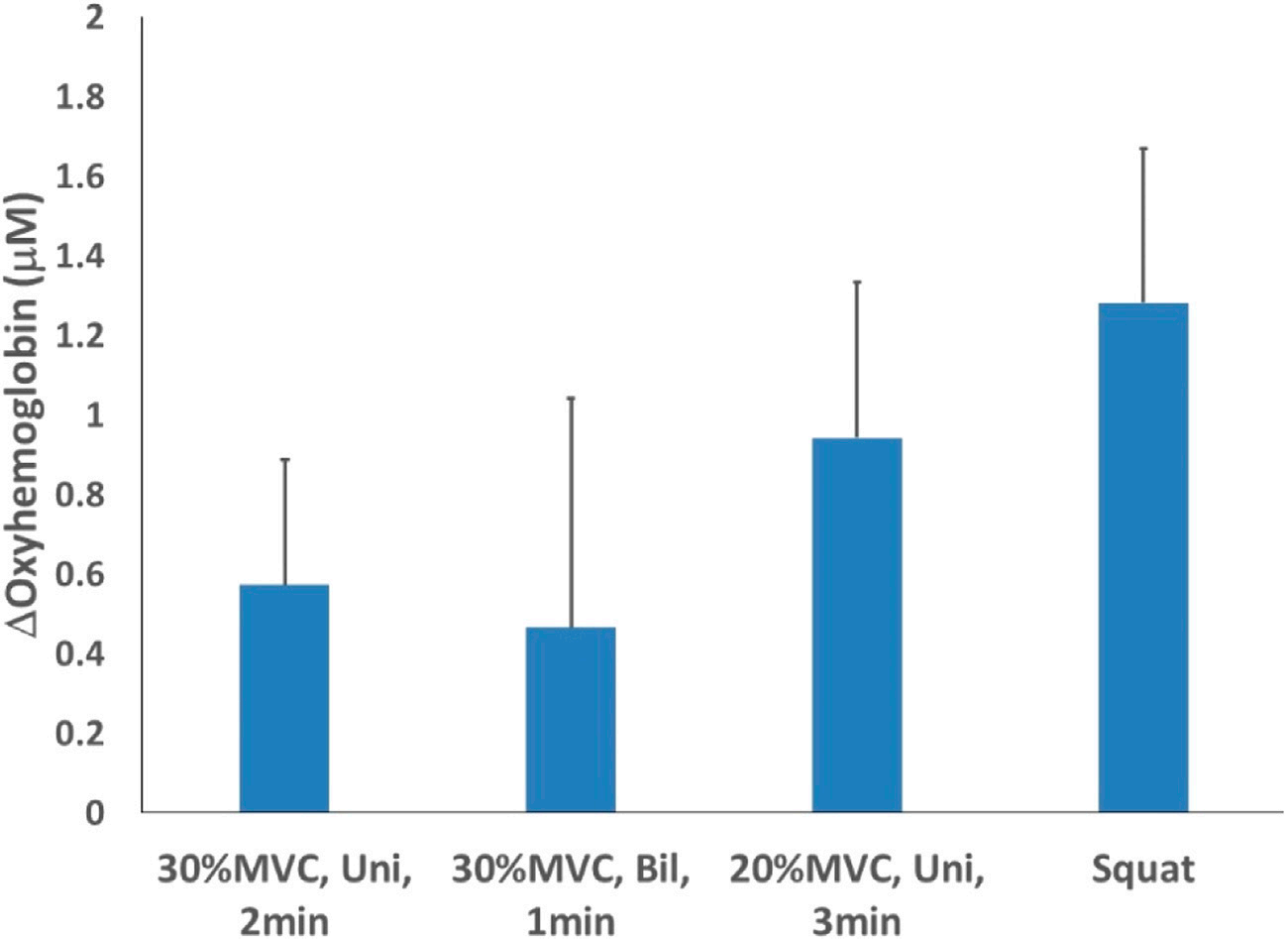
Oxyhemoglobin changes after 4 isometric exercise protocols.

For oxyhemoglobin, comparing the four test schemes with different intensity and duration, the isometric wall squat at 105–110° of knee angle for 1 min with 2-min rest for a total of 4 bouts (1.282 ± 0.389 μM), while the lowest mean was recorded for the bilateral isometric handgrip exercise at 30% MVC for 1 min with 2-min rest for a total of 4 bouts (0.466 ± 0.575 μM). Additionally, the unilateral isometric handgrip exercise at 30% of MVC for 2 min with 2-min rest for a total of 4 bouts (0.573 ± 0.314 μM) and the unilateral isometric handgrip exercise at 20% MVC for 3 min with 2-min rest for a total of 4 bouts (0.943 ± 0.389 μM) (Figure 2).

**FIGURE 3:**
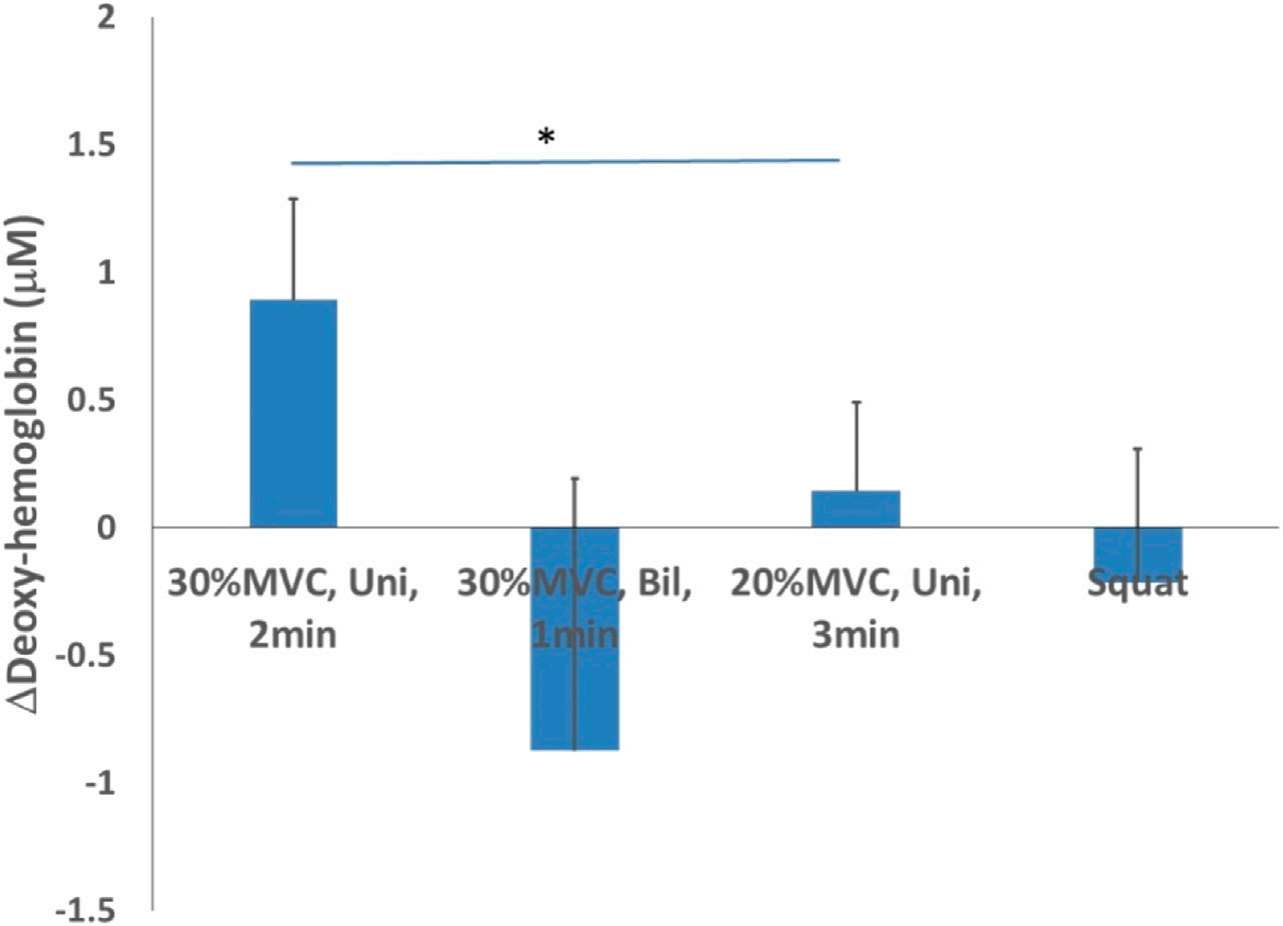
Deoxy-hemoglobin changes after 4 isometric exercise protocols.

When comparing the four test schemes with different intensity and duration for deoxy-hemoglobin, the highest mean was observed during the unilateral isometric handgrip exercise at 30% of MVC for 2 min with 2-min rest for a total of 4 bouts (0.890 ± 0.394 μM), while the lowest mean was recorded for the bilateral isometric handgrip exercise at 30% MVC for 1 min with 2-min rest for a total of 4 bouts (−0.873 ± 1.065 μM). Additionally, the unilateral isometric handgrip exercise at 20% MVC for 3 min with 2-min rest for a total of 4 bouts (0.144 ± 0.342 μM) and the isometric wall squat at 105–110° of knee angle for 1 min with 2-min rest for a total of 4 bouts (−0.214 ± 0.520 μM) (Figure 3).

**FIGURE 4:**
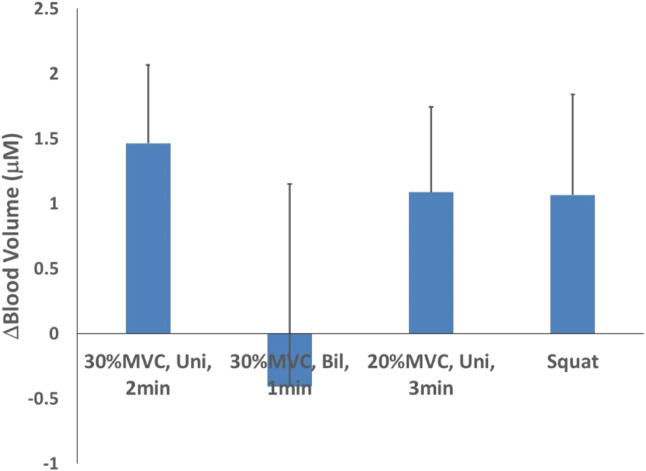
Blood volume changes after 4 isometric exercise protocols.

For blood volume, when comparing the four test schemes with different intensity and duration, the highest mean was observed for the unilateral isometric handgrip exercise at 30% of MVC for 2 min with 2-min rest for a total of 4 bouts (1.464 ± 0.605 μM), while the lowest mean was recorded for the bilateral isometric handgrip exercise at 30% MVC for 1 min with 2-min rest for a total of 4 bouts (−0.407 ± 1.557 μM). Additionally, the unilateral isometric handgrip exercise at 20% MVC for 3 min with 2-min rest for a total of 4 bouts (1.087 ± 0.658 μM) and the isometric wall squat at 105–110° of knee angle for the 1 min with 2-min rest for a total of 4 bouts (1.067 ± 0.772 μM) (Figure 4).

**FIGURE 5:**
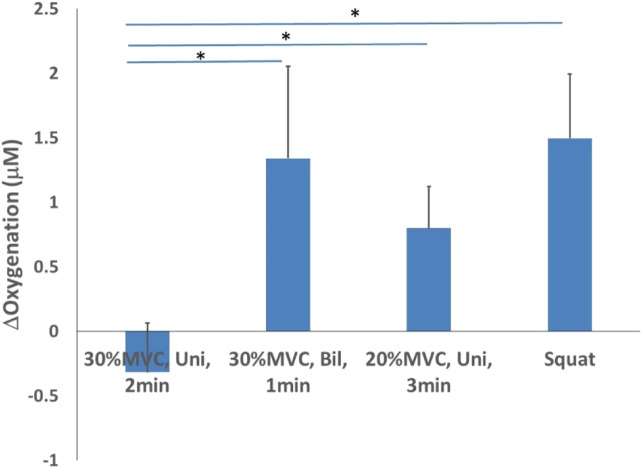
Oxygenation changes after 4 isometric exercise protocols.

For oxygenation, the lowest mean was recorded for the unilateral isometric handgrip exercise at 30% of MVC for 2 min (−0.317 ± 0.379 μM) while the highest mean was observed for the isometric wall squat (1.496 ± 0.498 μM, *p* < 0.05). Additionally, both the bilateral isometric handgrip exercise at 30% MVC for 1 min (1.340 ± 0.711 μM, *p* < 0.05) and the unilateral isometric handgrip exercise at 20% MVC for 3 min (0.798 ± 0.324 μM, *p* < 0.05) are significantly higher than 30% of MVC for 2 min (Figure 5).

**FIGURE 6:**
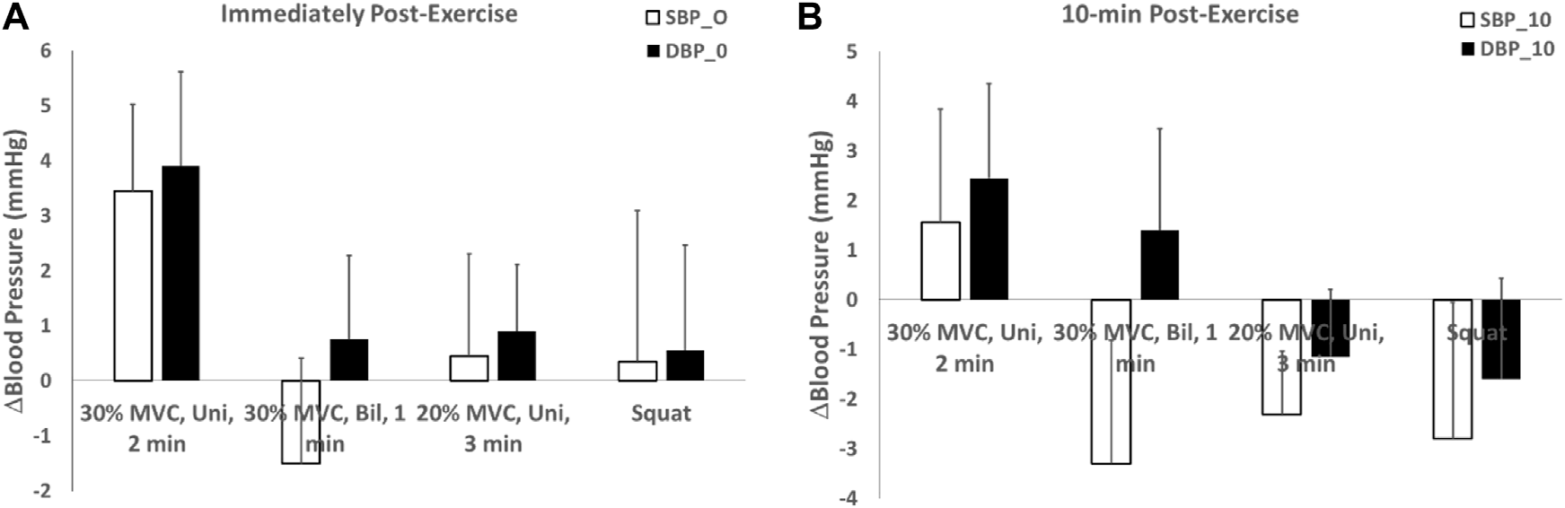
Systolic and diastolic blood pressure changes after isometric exercise **(A)** immediately after 4 isometric exercise protocols and **(B)** 10-min post-exercise.

For blood pressure immediately after isometric exercise, systolic blood pressure (SBP) and diastolic blood pressure (DBP) were measured. Among the four test schemes with different intensity and duration, the highest SBP (3.45 mmHg) and DBP (3.9 mmHg) was observed for the unilateral isometric handgrip exercise at 30% of MVC for 2 min with 2-min rest for a total of 4 bouts protocol. Additionally, unilateral isometric handgrip exercise at 20% MVC for 3 min with 2-min rest for a total of 4 bouts protocol with an SBP drop of 0.45 and a DBP drop of 0.9 mmHg, the isometric wall squat at 105–110° of knee angle for 1 min with 2-min rest for a total of 4 bouts protocol with an SBP drop of 0.35 and a DBP drop of 0.55 mmHg, bilateral isometric handgrip exercise at 30% MVC for 1 min with 2-min rest for a total of 4 bouts protocol with an SBP drop of −1.5 and a DBP drop of 0.75 mmHg (Figure 6A).

For blood pressure 10 min post-isometric exercise, both systolic and diastolic blood pressure decreased to varying degrees. Among the four test schemes with different intensity and duration, the unilateral isometric handgrip exercise at 30% of MVC for 2 min with 2-min rest for a total of 4 bouts protocol exhibited the smallest decrease in blood pressure, with a SBP drop of 1.55 mmHg and a DBP drop of 2.45 mmHg. Conversely, the isometric wall squat at 105–110 °ees of knee angle for 1 min with 2-min rest for a total of 4 bouts protocol showed the largest decrease in blood pressure, with an SBP drop of −2.8 and a DBP drop of −1.6 mmHg. Additionally, unilateral isometric handgrip exercise at 20% MVC for 3 min with 2-min rest for a total of 4 bouts protocol with an SBP drop of −2.3 and a DBP drop of −1.15 mmHg, bilateral isometric handgrip exercise at 30% MVC for 1 min with 2-min rest for a total of 4 bouts protocol with an SBP drop of −3.3 and a DBP drop of 1.4 mmHg. These results suggest that the isometric wall squats protocol is the most effective exercise for lowering blood pressure (Figure 6B).

## Discussion

This study demonstrates that the four isometric protocols of different intensities, durations, and muscle mass induced various degrees of changes in muscle hemodynamic responses and systematic blood pressure in healthy participants, and the post-exercise systematic blood pressure patterns are correlated with the change in local muscle oxygenation. The finding indicates that local muscle oxygenation induced by isometric exercise could affect systematic blood pressure regulation. A potential mechanism of action is autonomic nervous function that is responsible to regulate muscle blood flow and blood pressure (Millar et al., 2013). Our results suggest that isometric exercise of handgrip and wall squat changes local muscle oxygenation for reducing blood pressure. To the best of our knowledge, this is the first study exploring the relationship between muscle hemodynamic responses of isometric contraction and systematic blood pressure changes. Our finding provide foundation for determining the dose-response relationship of isometric contraction on blood pressure regulation.

Resting blood pressure after isometric exercise was found lower when measured 10-min post-exercise compared to immediately post-exercise. A potential mechanism mediating these changes is the central and peripheral autonomic regulation of vascular tone induced by isometric exercise (Yamada et al., 2022). Resting muscle sympathetic nerve activity is a marker of central efferent sympathetic outflow, and one study suggests that isometric handgrip strength training may decrease peripheral sympathetic vasoconstrictor activity and reduce increased sympathetic outflow in hypertensive patients. However, further studies are needed to determine whether peripheral sympathetic nerve activity could be added up by various factors (e.g., intensity, duration and muscle mass) of isometric exercise training (handgrip and wall squat in our case) in normotensive individuals (Ray and Carrasco, 2000; Yamada et al., 2022).

The results of the study demonstrated that bilateral isometric handgrip exercise at 30% MVC for 1 min (with 2-min rest for a total of 4 bouts) protocol had higher oxygenation values (1.340 ± 0.711 μM) immediately after isometric exercise compared to a common isometric exercise protocol, that is, unilateral isometric handgrip exercise at 30% of MVC for 2 min (with 2-min rest for a total of 4 bouts) (−0.317 ± 0.379 μM). These two protocols have the same intensity at 30% MVC but different durations and muscle mass, and the comparison of these two results could indicate that the efficacy of muscle mass on muscle oxygenation might be greater than the duration on oxygenation.This implies that the larger muscle mass involved in isometric exercise, the larger changes in local muscle oxygenation as well as systematic blood pressure. The central command theory involves the activation of higher brain centers at the onset of a motor control plan for executing muscle contraction (Galvez et al., 2000). It is reasonable to speculate that the greater the number of recruited motor units of the skeletal muscle to complete an isometric contraction task, the higher the integration of this signal by the central control neurons, the greater the amount of central command input to the cardiovascular center of the brainstem; and therefore, the greater the cardiovascular response after isometric exercise (Galvez et al., 2000). Surface EMG provides a way to measure motor unit recruitment and its discharge rate, and changes in intracellular metabolism of muscle fatigue can affect sEMG signals (Katayama et al., 2010). Several studies have reported the relationship between EMG and NIRS measures of muscle oxygenation during exercise (Amann et al., 2007; Yamada et al., 2008). Yamada et al. demonstrated that isometric contractions at 50% MVC showed changes in oxyhemoglobin and deoxy-hemoglobin that could indicate muscle fatigue as assessed by EMG (Yamada et al., 2008). Thus, the mutual regulation of central and peripheral sympathetic nerve activity during isometric exercise results in changes in blood pressure, as well as in muscle hemodynamic responses. In addition, during isometric contractions, elevated intramuscular pressure compresses the microcirculatory vasculature, resulting in sustained ischemia. Therefore, we suggest that muscle mass of isometric exercise may be an important factor on determining the changes in blood pressure after isometric exercise.

The results of the study demonstrated that unilateral isometric handgrip exercise at 20% MVC for 3 min protocol had higher oxygenation (0.798 ± 0.324 μM) than a common isometric exercise protocol, that is, unilateral isometric handgrip exercise at 30% of MVC for 2 min (−0.317 ± 0.379 μM) after isometric exercise. Comparisons between those two protocols with the same muscle mass involved in the exercise (unilateral handgrip for both protocols) indicate that the duration of isometric exercise has a greater effect on oxygenation than the intensity factor on oxygenation, and the longer the duration the higher the oxygenation values. A potential common mechanism of isometric exercise for lowering blood pressure and improving vascular endothelial function may be an increase in nitric oxide production and/or bioavailability (Beck et al., 2014). Increased nitric oxide at rest may reduce peripheral vascular resistance, which may contribute to lowering blood pressure. However, further studies are needed to investigate the role of vasoactive substances on the effects of resting blood pressure after isometric exercise. The results of two studies conducted by Wiley et al. with pre-hypertensive participants, which included an 8-week experiment with four 2-min isometric grip contractions at 30% MVC and a 5-week experiment with four 45 s isometric grip contractions at 50% MVC, showed similar eventual reductions in blood pressure. It suggests that lower isometric exercise intensities can be compensated by longer contraction times (Wiley et al., 1992).Therefore, our results suggest that the duration of isometric exercise might be a more important factor on modulating local muscle hemodynamics and systematic blood pressure responses than the intensity factor of isometric exercise. This finding is consistent with the literature (Taylor et al., 2003; Lawrence et al., 2015). Thus, this study supports the concept of isometric exercise duration dependence.

The results of the study showed that bilateral isometric handgrip exercise at 30% MVC for 1 min (with 2-min rest for a total of 4 bouts) (1.340 ± 0.711 μM) and isometric wall squats at 105–110° of knee flexion for 1 min (with 2-min rest for a total of 4 bouts protocol) (1.496 ± 0.498 μM) had higher oxygenation after isometric exercise compared to the other two handgrip protocols; and wall squats (lower limb) had the highest oxygenation values (1.496 ± 0.498 μM) compared to 3 handgrip protocols (upper limb). Our results indicate that muscle mass might be an important factor contributing to the different oxygenation values after isometric exercise of the upper *versus* lower extremities. The greater the amount of muscle mass involved in the isometric exercise, the higher the oxygenation values after the isometric exercise. The degree of adaptation to exercise stimuli is usually related to the mass of the tissue involved, and the degree of cardiovascular response to acute isometric contractions (i.e., systolic, diastolic, mean arterial pressure, and/or heart rate) has been reported to be positively correlated with an increase in muscle mass (Galvez et al., 2000). Healthy subjects performed isometric contraction with 50% and 20% of MVC and showed that the magnitude of the increase in the isometric movement center rate was related to the strength of the contraction and the quality of the contracted muscle (Galvez et al., 2000).

The results of the study showed that three protocols showed elevated oxygenation values measured 10-min post-exercise with decreased blood pressure values. Among them, isometric wall squats had the highest oxygenation value (1.496 ± 0.498 μM), and lowest blood pressure value. In addition, unilateral isometric handgrip exercise at 30% of MVC for 2 min (−0.317 ± 0.379 μM) showed a decrease in oxygenation values after isometric exercise, and its blood pressure measurement after 10 min showed an increase in blood pressure values, inhibiting the smallest decrease in blood pressure, with a SBP drop of 1.55 mmHg and a DBP drop of 2.45 mmHg. Alterations in vascular function are another potential mechanism for the reduction in resting blood pressure (Badrov et al., 2013). Research studies have shown that improvement in endothelial function occurs concomitantly with a reduction in diastolic blood pressure (Correia et al., 2020). Badrov et al. (2016) found that improvements in endothelial function were combined with reductions in resting systolic, diastolic, and mean blood pressures compared with baseline values (no control group comparison). Therefore, according to the results of this experiment, the changes in the blood pressure of the participants after isometric exercise showed an inverse relationship with the changes in oxygenation, which means that it can be predicted that the higher the value of oxygenation after isometric exercise, the greater the decrease in the final blood pressure value.

Our findings have clinical implications. Because our results show that the different intensity and duration of local muscle hemodynamic response caused by oxygenation value can predict the change of human blood pressure after exercise, (there is an inverse relationship, that is, the higher the oxygenation value, the more the human blood pressure decreased). However, it should be careful when applying our finding to hypertension patients. Our results also suggest that the duration of the isometric contraction and muscle mass factors can influence the oxygenation response and blood pressure regulation. Therefore, the oxygenation factors after isometric contraction may be a mediator of blood pressure regulation.

There are several limitations to this study. First, this study only investigated the acute response of local muscle hemodynamic response and blood pressure after isometric exercise, and it is unclear whether the long-term effects of isometric exercise at different intensities, durations and muscle mass are consistent on affecting the human muscle hemodynamic response and blood pressure. Secondly, this study was conducted in healthy adults and it is unclear whether our results could be applied to hypertensive patients. In the future, acute and long-term studies of muscle hemodynamic response and blood pressure can be conducted in patients with hypertension. Finally, this study did not conduct a separate and comprehensive analysis of the characteristics of the participant group (e.g., sex and age), and these individual components (e.g., participants’ emotion during 3 visits) are unlikely to operate in isolation but interact, so future studies may need to consider these common factors.

## Conclusion

This study investigated the acute effects of the intensity, duration, and muscle mass factors of isometric exercise on the local muscle hemodynamic response and systemic blood pressure regulation in healthy adults. Our results provide quantitative evidence that the duration of exercise has a greater effect on oxygenation under the same muscle mass involved in exercise, an isometric exercise protocol of the same intensity showed that muscle mass had a higher effect than the effect of duration on the oxygenation values. Of the four isometric exercise regimens of different intensity and duration, the highest after the 1-min exercise of the lower limbs, indicating that the muscle mass involved in isometric exercise had a large effect on the oxygenation value. In addition, the results showed an inverse relationship between muscle oxygenation values and blood pressure changes after isometric exercise. The findings of this study provide initial evidence between muscle hemodynamic responses of isometric contraction and systematic blood pressure regulation.

## Data Availability

The original contributions presented in the study are included in the article/supplementary material, further inquiries can be directed to the corresponding authors.
